# Role of MicroRNAs in acceleration of vascular endothelial senescence

**DOI:** 10.1016/j.bbrep.2022.101281

**Published:** 2022-05-26

**Authors:** Kensuke Toyama, Joshua M. Spin, Alicia C. Deng, Yasunori Abe, Philip S. Tsao, Masaki Mogi

**Affiliations:** a Department of Pharmacology, Ehime University Graduate School of Medicine, Ehime, Japan; b VA Palo Alto Health Care System, Palo Alto, CA, United States; c Division of Cardiovascular Medicine, Stanford University School of Medicine, Stanford, CA, United States

**Keywords:** microRNA, Cellular senescence, Vascular endothelial cells

## Abstract

**Backgrounds:**

Many factors are involved in cellular aging, and senescence induction requires complex regulation of various signaling networks and processes. Specifically, in the area of aging-related vascular cognitive impairment, laboratory-based findings have not yet yielded agents of practical use for clinical settings. One possible reason is that the physiologic elements of aging have been insufficiently considered. We sought to establish techniques to better model cellular aging using modulation of microRNAs, aiming to identify key microRNAs capable of fine-tuning aging-associated genes, and thereby regulating the senescence of vascular endothelial cells.

**Methods:**

We utilized expression microRNA arrays to evaluate control and senescent vascular endothelial cells in order to identify testable candidates. Bioinformatic analysis was used to select key microRNAs. These candidates were then modulated *in vitro* using microRNA mimics and inhibitors in endothelial cells, and senescence-associated gene expression patterns were evaluated by qPCR.

**Results:**

Seventeen microRNAs were found to be significantly increased more than 2-fold in senescent cells. Of those, bioinformatic analysis concluded that miR-181a-5p, miR-30a-5p, miR-30a-3p, miR-100-5p, miR-21-5p, and miR-382-5p were likely associated with regulation of cellular senescence. We evaluated the potential targets of these six microRNAs by comparing them with cell-cycling and apoptosis-related genes from published mRNA transcriptional array data from aged tissues, and found that miR-181a-5p, miR-30a-5p and miR-30a-3p were enriched in overlapping targets compared with the other candidates. Modulation of these microRNAs in vascular endothelial cells revealed that over-expression of miR-30a-5p, and inhibition of both miR-30a-3p and miR-181a-5p, induced senescence.

**Conclusion:**

miR-181a-5p, miR-30a-5p and miR-30a-3p likely contribute to aging-associated vascular endothelial cell senescence.

## Highlights

• We aimed to identify key microRNAs regulating the senescence of vascular ECs.
• Bioinformatic analysis indicated miR-181a-5p, miR-30a-5p & 30a-3p as candidates.
• Overexpression of miR-30a-5p & inhibition of miR-30a-3p/181a-5p induce EC senescence.

## 1 Introduction

Aging is the leading risk factor for numerous disease conditions. One of these, dementia, has become a progressively more prominent global problem in our aging society. The World Health Organization cautions that over 55 million people live with dementia worldwide, and that nearly 10 million new cases occur every year [1]. However, drug-based therapies for dementia have not been emerging as rapidly as expected, and investigations for novel candidates are required.

Recent work suggests that the proportion of patients with dementia related to vascular injury are nearly the same as the prevalence of Alzheimer's disease [2]. This suggests that vascular factors play an important role in cognitive pathology, and research interests are therefore shifting to focus on the role of blood brain barrier disruption in cognitive impairment [3,4]. However, as noted above, these investigations have not yet yielded practical applications for clinical settings. One probable reason is that the physiologic elements of aging (the primary background and major risk factor for dementia) have been insufficiently modeled in hypothesis-generating *in vitro* experiments.

Repeated passaging of cells, and cell-cycle arresting agents have been used to simulate cell-aging *in vitro* [5,6]. However, passaging can require a long time to induce an “aged cell” environment even in the most comprehensive cellular senescence models. On the other hand, drug-induced modification of the cell-cycle typically leads to cycle arrest, stressing the cells excessively despite being a more rapid method for mimicking a senescent environment. It is difficult to age primary human cerebral vascular endothelial cells for the experiments using standard methods because of their limited ability to survive *in vitro* [7]. It is also not simple to mimic tissue cellular senescence, since the process generally presents heterogeneously. Nonetheless, new time-saving but effective methods for simulating physiological cell-aging are needed. In particular, it would be helpful to establish such methods within an *in vitro*-blood brain barrier model of aging.

MicroRNAs, which can induce translational repression and degradation of multiple mRNAs, play key roles in various pathological conditions and developmental processes. Since these molecules can regulate multiple gene expressions at once, it is believed that microRNAs are central to fine-tuning gene regulation [8]. The aim of this study was to identify key microRNAs capable of simultaneous regulation of multiple aging-related genes, and to establish techniques for vascular endothelial cellular aging using microRNA modulation.

## 2 Materials and methods

### 2.1 Cell cultures

Human aortic endothelial cells (Lifeline Cell Technology; catalog number: KA-4009; Lot number: 02357) were cultured in collagen type I-coated dishes and grown in EGM-2 Complete Media (PromoCell GmbH; catalog number: C22111) at 37 °C in a 5% CO2 and 95% air humidified atmosphere.

### 2.2 Transfection of cultured cells

Endothelial cells were plated at 1.0 × 10^5^ cells per well (6 well plate; Nunc™ Poly-d-Lysine or Collagen I Coated Multidishes, Thermo Scientific™; catalog number: 152034), and transfection of anti-/pre-microRNAs initiated at ∼60% confluence. According to previous methods [4] with minor modifications, transfections of vascular endothelial cells were performed using Lipofectamine RNAiMAX transfection reagent (Invitrogen; catalog number: 13778) mixed with (1) anti-hsa-miR-30a-3p, anti-hsa-miR-30a-5p, and anti-hsa-miR-181a-5p (S-TuD; GeneDesign, Inc.), or (2) pre-hsa-miR-30a-3p, pre-hsa-miR-30a-5p, and pre-hsa-miR-181a-5p (mimic; GeneDesign, Inc.) or (3) scrambled controls with final concentrations of 10 nmol/L in serum-free medium (Opti-MEM; GIBCO; catalog number: 31985-070).

### 2.3 Animals

C57BL/6J mice were housed in a temperature-controlled (20 ± 2 °C) and humidity-controlled (60%) room under a 12 h light/dark cycle (8:00/20:00). Experimental protocols were approved by the Committee for Laboratory Animal Care and Use in Ehime University (approval number; 05KI31-1). All procedures were in accordance with institutional guidelines for the care and use of laboratory animals.

### 2.4 Quantitative real-time polymerase chain reaction (qPCR)

ReverTra Ace qPCR RT Master Mix (TOYOBO; catalog number: FSQ-201) was used to synthesize first-strand cDNA from mRNA. QPCR assays were performed using the qTOWER³ G (Analytik Jena GmbH). THUNDERBIRD SYBR qPCR Mix (TOYOBO; catalog number: QPS-201) was used for amplifications. Specific oligonucleotide primers for target sequences are shown in Supplementary Table 1. GAPDH was used as an internal control for normalization. All fold changes were calculated by the method of ΔΔCt.

### 2.5 Senescence-associated-β-galactosidase (SA-β-gal) staining

According to the manufacturer's protocol, SA-β-gal staining was performed on cell culture dishes using a Cellular Senescence Kit (OZ Biosciences Inc.; catalog number: GXS0003). After taking at least five photos per well at random locations, numbers of SA-β-gal positive cells were counted to obtain the percentage of senescent cell density against total cells using ImageJ software.

### 2.6 Bioinformatics analysis

DIANA-mirPath V.3 analysis [9] was used to predict target genes of differently expressed microRNAs. To more specifically narrow down potential target genes of microRNAs, the list of predicted target genes was integrated with the differentially expressed genes (DEGs) of GEO datasets GSE9990 [10] and GSE53890 [11]. Since our future aim is to more closely mimic the blood brain barrier in a cultured senescence cell model, we utilized transcriptional profiles of (1) rodent hippocampal tissues with cognitive decline (GSE9990 dataset; 3 months [n = 9] vs. 23 months old samples [n = 13]) and (2) aged human brain samples from frontal cortical regions (GSE53890 dataset; <40 years old [n = 12] vs. 70–94 years old samples [n = 16]) for this analysis.

### 2.7 MicroRNA array analysis and statistical analysis

RNA samples were analyzed using the Gene Chip miRNA 4.0 Array (Affymetrix, CA, USA). Replicate microRNAs were averaged, and the expression data were normalized. Scatter plotting was used to identify significantly differentially expressed microRNAs. Statistical testing (*t*-test) and visualization of differentially expressed microRNAs were all performed using Transcriptome Viewer software (Kurabo Industries Ltd., Osaka, Japan). Signal numbers were normalized by the RMA-Sketch algorithm and are log base 2. Statistically significant differentially expressed microRNAs were defined as P < 0.05 and | log_2_ Fold Change |> 1.0.

The relationships of gene expression changes between the groups were assessed using linear regression analysis (Pearson correlation test). To compare the difference of linear regressions between groups, William's test was performed using R version 4.1.3 (The R Foundation for Statistical Computing, Vienna, Austria).

## 3 Results

**Fig. 1:**
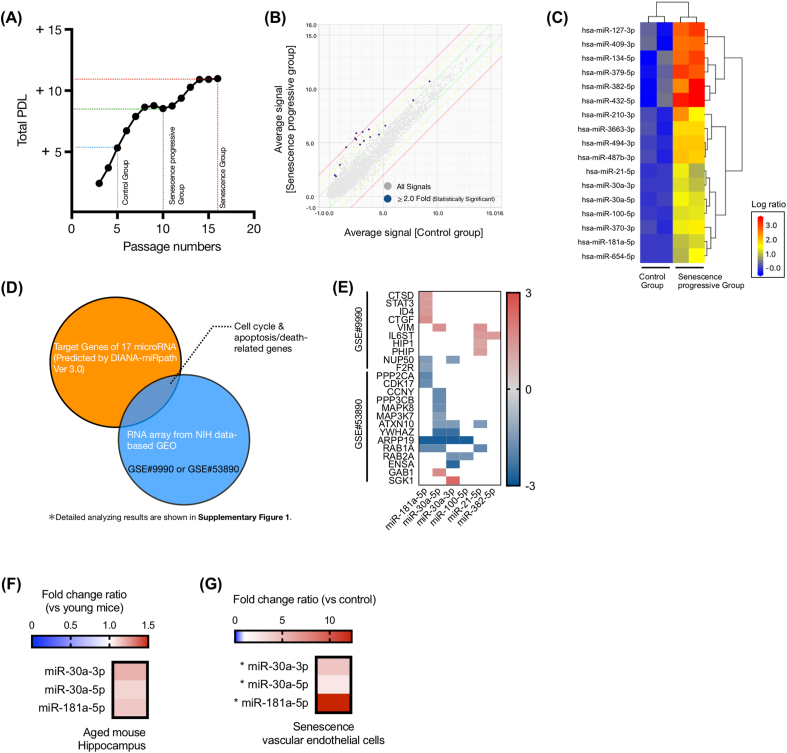
(A) Change of population doubling level (PDL) graphed against the passage numbers of human aortic endothelial cells. (B) Scatter plot showing differentially expressed microRNAs in aging cells versus control cells (n = 2 in each group). (C) Expression level (log2) heat map of the differentially expressed candidate microRNAs between control and aging cells (n = 2 in each group). (D) Venn diagram: Target genes of 17 microRNAs which were predicted from bioinformatic analysis (orange color), and up- or down-regulated genes in aging brain tissue (derived from RNA array results; blue color). (E) Raw gene expression levels which were derived from (D) shown against their targeting microRNAs. (F) Fold change ratios of miR-30a-3p /miR-30a-5p /miR-181a-5p in C57BL/6 aged-mouse hippocampus (108-113 weeks-old; n = 6) graphed against control young mouse hippocampus (13-15 weeks-old; n = 6). (G) Fold change ratios of miR-30a-3p /miR-30a-5p /miR-181a-5p in the senescence vascular endothelial cells (n = 4) against control cells (n = 4). * indicates statistical significance compared to control. (For interpretation of the references to color in this figure legend, the reader is referred to the Web version of this article.)

It is difficult to age primary human cerebral vascular endothelial cells due to their limited survival *in vitro*. Accordingly, we used primary human aortic endothelial cells instead to model cellular senescence. To evaluate microRNA changes with senescence in these vascular endothelial cells, they were passaged until they stopped dividing. Cultured cells were defined as “senescent” if they failed to reach confluency in one week (passage-16; red line in Fig. 1A). In Fig. 1A, the population doubling level (PDL - refers to the total number of times the cells in the population have doubled) is graphed against the cell passage count, and shows plateauing with senescence. In an effort to capture gene regulation during the process of cellular senescence rather than at the conclusion, cells that reached medium-passage #’s (passage-10; green line in Fig. 1A: “senescence progressive group”) were used for analysis instead of end-state senescent cells (red line). Passage-5 cells were used as the control group (blue line in Fig. 1A).

Scatter plot analysis of differentially expressed microRNAs between the cellular senescence progressive and control groups is shown in Fig. 1B. Expression levels > 2-fold and <0.5-fold are shown above or below the green lines. The expression levels of human microRNAs with P < 0.05 and | log2 Fold Change |> 1.0 are shown as blue dots. Expression data for each blue dot microRNA are detailed in Supplementary Table 2. A total of seventeen microRNAs showed statistically significant increased expression compared to the control group. A heatmap of the expression levels for these seventeen microRNA is shown in Fig. 1C (hsa-miR-100-5p, miR-181a-5p, miR-210-3p, miR-370-3p, miR-654-5p, miR-382-5p, miR-494-3p, miR-487b-3p, miR-432-5p, miR-134-5p, miR-379-5p, miR-3663-3p, miR-127-3p, miR-409-3p, miR-30a-5p, miR-21-5p, and miR-30a-3p).

We utilized the DIANA-mirPath V.3 analysis tool [9] to extract cell cycle and apoptosis/death-related genes which are predicted to be targeted by each of the seventeen microRNAs (Fig. 1D; orange circle). Predicted target-gene lists are shown in Supplementary Tables 3–10. Since the aim of the present study was to find key microRNAs capable of simulating aging of the blood-brain barrier, we next obtained genes (top hit genes <250) from two relevant public NCBI-GEO datasets (Fig. 1D; blue circle): (1) gene expression in aged human brain cortex (GEO accession number: GSE9990 [10]), and (2) aged rat hippocampus (GEO accession number: GSE53890 [11]). Supplementary Fig. 1 shows a detailed analysis of the results of Fig. 1D.

Ultimately, there were 10 candidate target genes (cathepsin D [CTSD], signal transducer and activator of transcription 3 [STAT3], inhibitor of DNA binding 4 [ID4], connective tissue growth factor [CTGF], vimentin [VIM], interleukin 6 cytokine family signal transducer [IL6ST], huntingtin interacting protein 1 [HIP1], pleckstrin homology domain interacting protein [PHIP], nucleoporin 50 [NUP50], and coagulation factor II thrombin receptor [F2R]), that were extracted from overlapping gene lists between the DIANA based-group (from the 17 microRNA-target sets) and the GSE #9990 based-group (Supplementary Fig. 1). An additional 14 candidate genes were extracted from the overlap between the DIANA based-group and the GSE #53890 based-group: protein phosphatase 2 catalytic subunit alpha [PPP2CA], cyclin dependent kinase 17 [CDK17], cyclin Y [CCNY], protein phosphatase 3 catalytic subunit beta [PPP3CB], mitogen-activated protein kinase 8 [MAPK8], mitogen-activated protein kinase kinase kinase 7 [MAP3K7], ataxin 10 [ATXN10], tyrosine 3-monooxygenase/tryptophan 5-monooxygenase activation protein zeta [YWHAZ], cAMP-regulated phosphoprotein 19 [ARPP19], ras-related protein rab-1A [RAB1A], ras-related protein rab-2A [RAB2A], endosulfine alpha [ENSA], growth factor receptor bound protein 2-associated protein 1 [GAB1], and serum/glucocorticoid regulated kinase 1 [SGK1]) (Supplementary Fig. 1).

Based on these candidates, 6 total microRNAs (miR-181a-5p, miR-30a-5p, miR-30a-3p, miR-100-5p, miR-21-5p, and miR-382-5p) emerged as likely regulators of cell-cycle-related genes (gray-shaded sets in Supplementary Fig. 1). Fig. 1E shows the expression heatmap of the collected set of 24 genes in the public datasets, matched to their predicted targeting microRNAs. Notably a subset of the microRNAs: miR-181a-5p, miR-30a-5p and miR-30a-3p, were predicted to target considerably more of the 24 gene subset when compared with the other candidate microRNAs, and therefore these three were chosen for additional studies.

MiR-30a-3p, miR-30a-5p and miR-181a-5p were increasing in aged mouse hippocampus compared to young mouse hippocampus (Fig. 1F). Further, senescence vascular endothelial cells (passage 16) demonstrated significantly elevated expression levels of these microRNAs (Fig. 1G). These results suggest that the three microRNAs might be increased in-common between aged brain vascular endothelial cells and the senescence vascular endothelial cells.

**Fig. 2:**
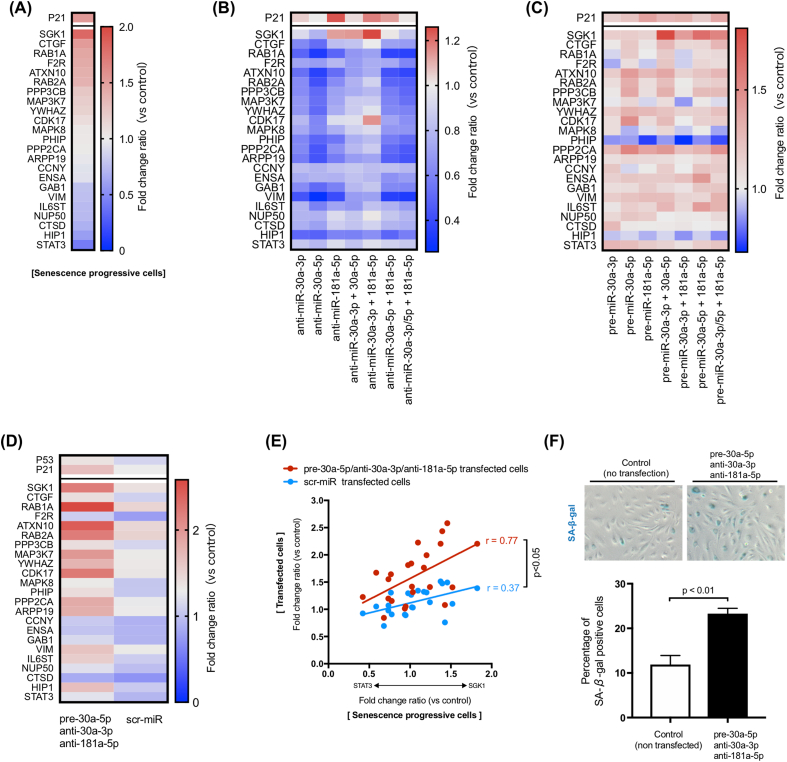
(A) Gene expression patterns of senescence progressive vascular endothelial cells (n = 7; and, control cells; n = 8). Gene expression patterns in (B) miR-30a-3p /miR-30a-5p /miR-181a-5p overexpressed and (C) miR-30a-3p /miR-30a-5p /miR-181a-5p under-expressed vascular endothelial cells (n = 3 in each group). (D) Changes of gene expression levels between scrambled-microRNA and pre-miR-30a-5p/anti-miR-30a-3p/anti-miR-181a-5p transfected vascular endothelial cells (vs. non-transfected control cells, n = 6; or scrambled-miR transfected cells, n = 4; pre-miR-30a-5p/anti-miR-30a-3p/anti-miR-181a-5p transfected cells, n = 5). (E) Percentage of SA-β-gal positive cells in the pre-miR-30a-5p/anti-miR-30a-3p/anti-miR-181a-5p transfected vascular endothelial cells (n = 4 in each group).

Fig. 2A shows expression of the targeted geneset in the senescent cells. ID4 gene expression was extremely low in the vascular endothelial cells, and therefore is not shown in this heatmap. Fig. 2B and C respectively show the expression for the gene set when hsa-miR-181a-5p, miR-30a-5p and miR-30a-3p were intentionally over- and/or under-expressed individually or in combination (seven patterns were used in total). As shown in Fig. 2B, SGK1 showed increased expression with only three combinations (anti-miR-181a-5p alone, anti-miR-30a-3p + anti-miR-30a-5p, and, anti-miR-30a-3p + anti-miR-181a-5p), similar to its expression direction in senescence-trending cells. Also, only the combination of anti-miR-30a-3p and anti-miR-181a-5p transfection increased expression of CDK17. Further, overexpression of miR-30a-5p (Fig. 2C) using pre-miR-30a-5p increased expression of several genes shown to be upregulated with senescence (from CTGF to YWHAZ – see parallel Fig. 2A). These results suggested that the most effective combination would be pre-miR-30a-5p, anti-miR-30a-3p and anti-miR-181a-5p.

When this combination was used in vascular endothelial cells, the gene expression pattern came close to approximating that seen in replicative-senescence cells (Fig. 2D). As shown in Fig. 2A, gene expression was decreased in order from SGK1→STAT3 in the senescence profile cells. We evaluated the linear regression of gene expression changes between the pre-miR-30a-5p/anti-miR-30a-3p/anti-miR-181a-5p- and scrambled microRNA-transfected cells, and compared them against those of the senescence progressive cells. Gene expression changes of senescence progressive cells correlated significantly more with those of pre-miR-30a-5p/anti-miR-30a-3p/anti-miR-181a-5p transfected cells (red line) than with those of scrambled microRNA (scr-miR; negative control)-transfected cells (blue line) (Fig. 2E). This indicates that pre-miR-30a-5p/anti-miR-30a-3p/anti-miR181a-5p transfected cells appear to be mimicking a senescent cell gene pattern. Further, gene expression of p21 and p53 (well known as aging markers) increased significantly more with miR-modulation than with scr-miR-transfected cells (Fig. 2D). Interestingly, expression of IL6ST, an inflammation-related gene, was elevated in transfected cells (Fig. 2D). This suggests the cells are manifesting the “senescence-associated secretory phenotype” [12]. Finally, we stained for SA-β-gal (one of markers of senescence) in the pre-miR-30a-5p/anti-miR-30a-3p/anti-miR-181a-5p transfected vascular endothelial cells (Fig. 2F), and found more SA-β-gal positive cells in the transfected cells than in the control cells.

## 4 Discussion

The present investigation shows that simultaneous upregulation of miR-30a-5p, and downregulation of both miR-30a-3p and miR-181a-5p, can mimic induction of cellular senescence in vascular endothelial cells. Such induction can be performed in a single step, as opposed to the time-consuming process of replicative passage senescence.

It is difficult to perfectly model the aging environment *in vitro* and *in vivo*. Of the most commonly used aging organism models (*Saccharomyces cerevisiae, Caenorhabditis elegans, Drosophila melanogaster*, and *Mus musculus*), mouse is the most often used for various reasons, particularly because of their biological similarity to humans [13]. Although elderly mice are used for research, it can take years to get them to the appropriate age. Genetically modified mice, such as accelerated-aging mice, have been developed and used as well [14].

*In vitro,* there are several cell models mimicking cell senescence. Researchers often create an aged-cell culture model by repeatedly passaging cells. In general, cultured cells are defined as senescent if they fail to reach confluency for an extended period after many passages [5]. However, this method is time-consuming since it requires numerous population doublings for cells to reach senescence [5]. Small-molecule MDM2 inhibitors like nutlin-3a, which inhibits the interaction between p53 and MDM2, are also used to mimic cell senescence [6], although such aggressive cell-cycle arrest seems less likely to accurately simulate aging. Cellular senescence is one of the elements contributing to organismal aging, and is triggered by multiple factors such as DNA damage, telomere attrition, and various epigenetic alterations, and does finally evolve into permanent cell-cycle arrest [13]. In these experiments we sought to find key factors involved in senescence-related cell cycle dysregulation.

The microRNAs identified in the present study have verified targets that might contribute in addition to the ones noted here. MiR-30a, including both miR-30a-3p and miR-30a-5p, has been reported to act as a tumor suppressor in various cancers. In osteosarcoma cells, under-expression of miR-30a-3p decreases the expression of “phosphatase and tensin homologue deleted on chromosome 10” (PTEN), known as one of the tumor suppressor genes [15]. MiR-30a-3p also has an inhibitory effect on growth and migration in gastric cancer cells through targeting of the cyclooxygenase-2 gene [16]. Similarly, miR-30a-5p inhibits the proliferation, migration and invasion of melanoma cells via regulation of “sex determining region Y-box 4 gene” [17]. Given that both miR-30a-3p and miR-30a-5p seem to be involved with suppressing cell proliferation, it is interested that inverse directions of modification (under-expressing miR-30a-3p and over-expressing miR-30a-5p) seemed to induce cellular senescence in the present work.

The miR-181 family regulates many biological processes, including cell proliferation, apoptosis, mitochondrial function, and immune responses [18]. MiR-181a plays an important role in cellular senescence via the downregulation of Sirtuin-1 [19], a well-known gene which opposes senescence [20]. Further, miR-181a is increased in patients with mild cognitive impairment [21]. Although miR-181a is thought of as a senescence-accelerating microRNA, our present work concluded that suppression of miR-181a-5p (when combined with inhibition of miR-30a-3p and over-expression of miR-30a-5p) may contribute to cellular senescence.

Recent studies have revealed that an endothelial progenitor cell (EPC) population is located at the inner surface of blood vessels (another term is “endothelial colony forming cells”) [22]. Senescent EPCs contribute less to vascular repair and regeneration. MiR-10a-3p, miR-21-5p and miR-22-5p can regulate EPC senescence by suppressing expression of high-mobility group A2 and AKT serine/threonine kinase 3, respectively [23,24]. MiR-34a-5p, a well-known aging microRNA, has also been reported to be involved with endothelial progenitor cell senescence [25]. It is possible that the microRNAs we identified might also be associated with EPC senescence *in vitro*, although further investigations are clearly required to clarify this.

There are several limitations in the current study. We would have preferred to use primary human cerebral vascular endothelial cells for our experiments, but it is difficult to age these cells using standard methods. If our microRNA-based approach is successful, aging experiments on human cerebral vascular endothelial cells might become more straightforward. Notably the definition of aging at the cellular level is also difficult. While gene expression showing elevation of p21 and p53, and SA-β-gal activity usually are used to define cellular aging, one may not simply conclude that cells showing these changes are physiologically “aged”. Further investigations are needed to overcome these issues.

## Funding sources

This study was partially supported by (1) Grant-in-Aid for Young Scientists (Start-up: JSPS KAKENHI Grant Number: 18H06213 & 19K21316; to Toyama K), (2) Grant-in-Aid for Scientific Research (C) (JSPS KAKENHI Grant Number: 20K07805; to Toyama K) and (3) 10.13039/100007434Suzuken Memorial Foundation (to Toyama K).

## Declaration of competing interest

The authors declare that they have no conflict of interest.

## Data Availability

Data will be made available on request.
