# Supplementary figures and images for: Role of MicroRNAs in acceleration of vascular endothelial senescence
Source: Biochem Biophys Rep. 2022 May 26;30:101281. doi: 10.1016/j.bbrep.2022.101281 (PMC9149016; doi:10.1016/j.bbrep.2022.101281)

Supplementary Figure 1

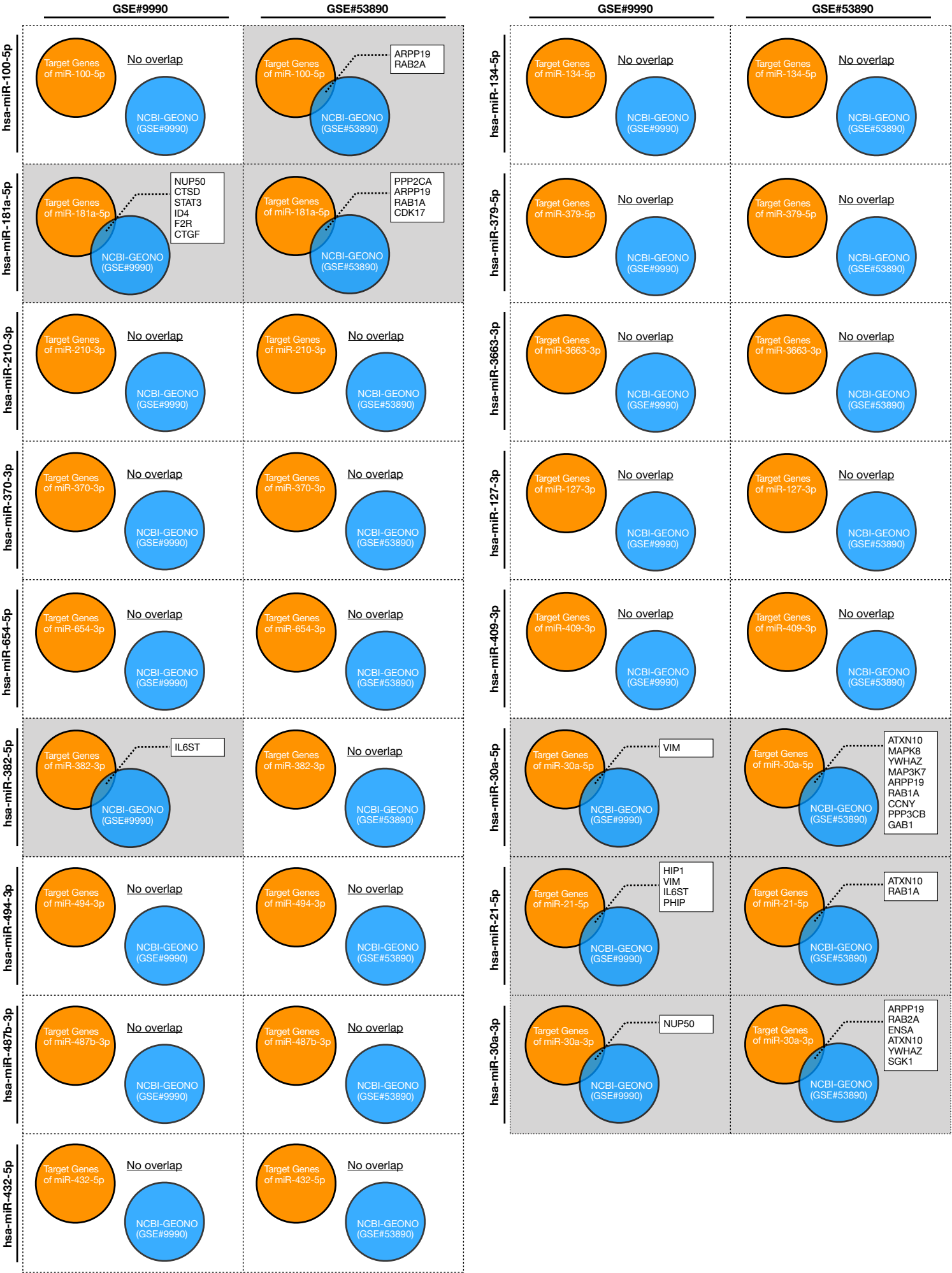

Supplement: Multimedia component 1 [file mmc1.pdf]
